# Non-Structural Protein V of Canine Distemper Virus Induces Autophagy via PI3K/AKT/mTOR Pathway to Facilitate Viral Replication

**DOI:** 10.3390/ijms26010084

**Published:** 2024-12-25

**Authors:** Xin Tian, Rui Zhang, Shuang Yi, Yu Chen, Ying Jiang, Xianwen Zhang, Zhidong Zhang, Yanmin Li

**Affiliations:** Key Laboratory of Veterinary Medicine in Universities of Sichuan Province, College of Animal Husbandry and Veterinary Medicine, Southwest Minzu University, 16 Yihuan Rd., Chengdu 610041, China

**Keywords:** canine distemper virus (CDV), CDV-V, PI3K, autophagy

## Abstract

Canine distemper (CD) is a highly infectious disease of dogs which is caused by canine distemper virus (CDV). Previous studies have demonstrated that CDV infection can induce autophagy in cells. However, the mechanism underlying CDV-induced autophagy remains not fully understood. The CDV non-structural protein V plays a vital role in viral replication and pathogenicity in the host. In this study, we investigated the relationship between the CDV-V protein and autophagy induction and further explored its impact on the viral replication and the mechanism behind this. Our results showed that the V protein induced autophagy via inhibiting the phosphorylation of PI3K, AKT, and mTOR to promote viral replication. The activation or inhibition of PI3K phosphorylation resulted in enhancing or reducing viral replication, respectively. Further studies revealed that the V protein interacted with PI3K to induce cellular autophagy. The present study demonstrated that the CDV-V protein can induce cellular autophagy by inhibiting the PI3K/AKT signaling pathway to enhance viral replication. The results improve the understanding of the molecular mechanism of CDV infection and offer new perspectives for the development of effective treatment and prevention strategies.

## 1. Introduction

Canine distemper (CD) is an acute, febrile, high-contact, infectious disease that affects canines, skunks, and some raccoons. It is caused by canine distemper virus (CDV) [1], which belongs to the genus *Morbillivirus* in the *Paramyxoviridae* family. Its genome length is about 15,616 bp and consists of six genes, namely the N, P, M, F, H, and L genes from the 3′ end to the 5′ end, which encode six structural proteins, respectively: nucleoprotein, phosphoprotein, matrix protein, fusion protein, hemagglutinin protein, and large protein [2]. Additionally, two non-structural proteins—namely C and V proteins—are encoded by the P gene. Many members of the paramyxovirus family encode C protein via the second ATG (start codon) within the open reading frame (ORF) of the P gene [3]. The V protein shares the start codon of the ORF of the P protein, and insertion of a G into the conserved RNA editing site results in the production of the V protein. In the absence of base insertion at this site, the P protein is synthesized. The V protein serves as a virulence factor for viruses of the genus paramyxovirus [4,5].

Non-structural proteins V and C are associated with viral evasion of host immunity during viral replication. Among them, the V protein is a key protein in the anti-host interferon response, especially in the early stages of viral infection [6]. The V protein is able to bind to the MDA5 molecule, block the IFN-α and IFN-β signaling pathways, disrupt JAK-STAT signaling, and prevent the formation of STAT protein dimers and nuclear localization effects, as well as cause STAT1 and STAT2 phosphorylation [7,8]. Meanwhile, it has been demonstrated that CDV causes apoptosis, inflammation, and autophagy while breaking through innate immunity to achieve self-replication [9,10,11]. The V protein plays an important role in the regulation of immune activity, and it remains unclear whether there are other immunological functions apart from regulating the antiviral activity of IFN.

When cells are exposed to environmental stresses such as infection with pathogenic microorganisms, nutritional deficiencies, and hypoxia, they undergo a series of signaling to form a self-protective mechanism that induces autophagy [12]. Phosphatidylinositol 3-kinase (PI3K)/AKT/mTOR is an important intracellular signaling pathway. PI3K can be classified into three categories according to its structure and function. Among them, type I is the most widely studied; it consists of the regulatory subunit p85 and the catalytic subunit p110 and is divided into two subfamilies, IA and IB [13]. Activation of PI3K leads to the conversion of phosphatidylinositol 4,5-bisphosphate (PIP2) to phosphatidylinositol 3,4,5-trisphosphate (PIP3), able to recruit 3-phosphoinositide-dependent kinase 1 (PDK1) onto membranes. Subsequently, PDK1 phosphorylates downstream AKT to complete its activation [14]. mTOR acts as an important downstream signaling node for AKT. AKT can indirectly activate mTOR by directly phosphorylating and inhibiting the TSC complex, thereby eliminating its inhibitory effect on mTOR [15,16]. In addition, AKT can also directly activate mTOR by phosphorylating and inhibiting PRAS40 (a component of mTOR that negatively regulates the kinase activity of the complex), which activates a series of cascading effects to inhibit autophagy [17].

Although autophagy is the basic cellular defense mechanism against viral invasion, many viruses can induce the onset of cellular autophagy and use the autophagy mechanism to promote viral replication, including foot-and-mouth disease virus [18], hepatitis B virus [19], pseudorabies virus [20], and porcine epidemic diarrhea virus [21]. Our recent study has found that CDV infection induces autophagy and promotes viral replication [10], yet the mechanism by which it induces autophagy remains unclear.

The CDV-V protein is a crucial component in the virus’s evasion of host immunity and its replication process. This study will focus on elucidating the role of the CDV-V protein in inducing autophagy in cells and will further investigate the molecular mechanisms by which the V protein induces autophagy. This study is aimed at enhancing our understanding of the pathogenesis of CDV and provides scientific insights into effective prevention and treatment strategies for CDV infection.

## 2. Results

### 2.1. CDV-V Protein Triggers Autophagy

**Figure 1:**
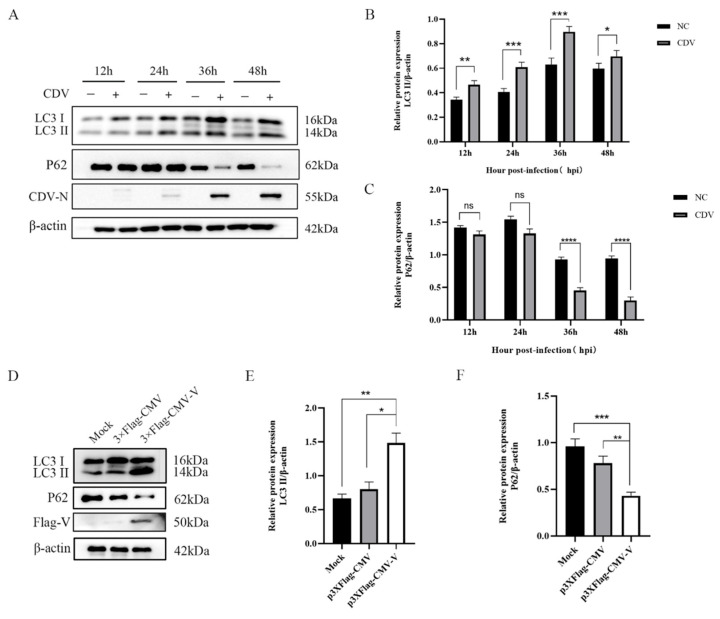
CDV-V induces cellular autophagy. (**A**) After 12 h, 24 h, 32 h, and 48 h of CDV (MOI = 1) infection. The cell samples were then analyzed using Western blot with anti-LC3B, anti-P62, anti-CDV-N, and anti-β-actin antibodies. (**B**) LC3. (**C**) P62 levels relative to the β-actin levels were determined by densitometry. (**D**) Vero cells were transfected with either p3 × Flag-CMV or p3 × Flag-CMV-V plasmids for 24 h. The expression levels of LC3B, P62, Flag-V, and β-actin were detected by Western blot. (**E**) The relative levels of LC3B were normalized to β-actin levels using densitometry. (**F**) The relative levels of P62 were normalized to the β-actin levels by densitometry. The data represent the mean ± SD of three independent experiments. Two-way ANOVA; * *p* < 0.05; ** *p* < 0.01; *** *p* < 0.001; **** *p* < 0.0001.

To verify whether CDV infection can induce autophagy, the level of autophagic marker protein LC3 (microtubule-associated protein1 light chain 3) was monitored in Vero cells using Western blotting. As shown in Figure 1A,B, the level of LC3-II increased as CDV infection progressed, with the most significant increase in autophagy at 36 h post-infection (hpi), which was consistent with the accumulation of CDV N protein. To assess whether CDV-induced autophagy is fully executed, we examined the degradation of polyubiquitin-binding protein p62/SQSTM1 (sequestosome 1), which is involved in the autophagy-mediated protein degradation pathway. The results showed no significant P62 expression changes at 12 and 24 hpi; however, a decrease in p62 levels occurred at 36 and 48 hpi, indicating that CDV induced a complete autophagic process (Figure 1A,C).

The non-structural protein V of CDV plays a pivotal role in viral replication. To explore its potential role in autophagy induced by CDV infection, Vero cells were transfected with the recombinant plasmid encoding the V protein tagged with Flag and were harvested at 24 h post-transfection for Western blot analysis. The results showed an increase in the LC3-II expression level and a decrease in the p62 expression level in the transfected cells, indicating that the CDV-V protein triggers autophagy (Figure 1D,E).

### 2.2. CDV-V Induces Autophagy via Inhibiting PI3K–AKT–mTOR Pathway

**Figure 2:**
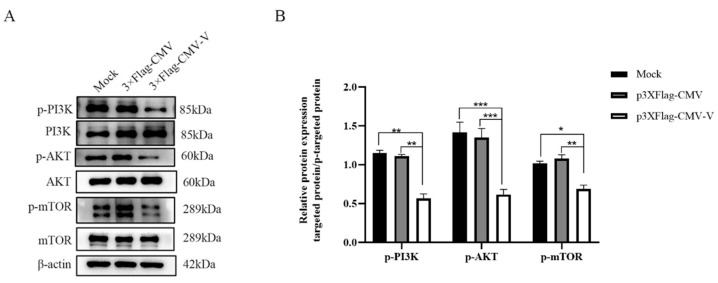
CDV-V induces autophagy through the PI3K/AKT signaling pathway. (**A**) Vero cells were transfected with either p3 × Flag-CMV or p3 × Flag-CMV-V plasmids for 24 h. The cell samples were then analyzed by Western blot with anti-p-PI3K, anti-PI3K, anti-p-AKT, anti-AKT, anti-p-mTOR, anti-mTOR, and anti-β-actin antibodies. (**B**) The ratios of p-PI3K/PI3K, p-AKT/AKT, and p-mTOR/mTOR were quantified by densitometry. The data represent the mean ± SD of three independent experiments. Two-way ANOVA; * *p* < 0.05; ** *p* < 0.01; *** *p* < 0.001.

The PI3K/AKT/mTOR pathway is a classic pathway that regulates cellular autophagy. To determine whether autophagy induced by CDV-V in Vero cells is regulated by this pathway, we examined the phosphorylation levels of PI3K, AKT, and mTOR by Western blot. As shown (Figure 2A,B), the phosphorylation levels of these proteins were significantly reduced in the cells transfected with CDV-V compared to the control cell group. This suggests that the PI3K/AKT/mTOR pathway is involved in V-protein-induced autophagy.

**Figure 3:**
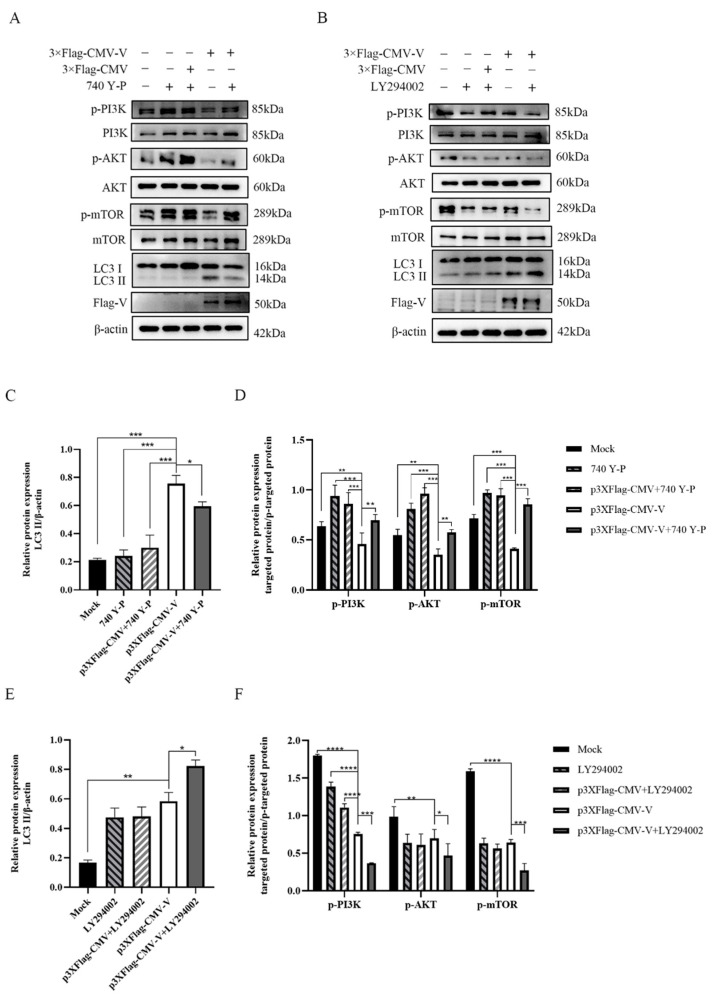
Effect phosphorylation of PI3K activation and inhibition on autophagy. (**A**) Based on the references, Vero cells were pretreated with 15 μM of 740 Y-P for 3 h before transfection [22], and then cell samples were analyzed by Western blot using anti-p-PI3K, anti-PI3K, anti-p-AKT, anti-AKT, anti-p-mTOR, anti-mTOR, anti-LC3B, anti-Flag, and anti-β-actin antibodies. (**B**) Vero cells were treated with 10 μM LY294002 for 1 h before transfection [23], then post-processing was the same as (**A**). (**C**) The ratio of p-PI3K/PI3K, p-AKT/AKT, and p-mTOR/mTOR was quantitated by densitometry. (**D**) LC3 levels relative to the β-actin levels were determined by densitometry. (**E**) Quantitative analysis of p-PI3K/PI3K, p-AKT/AKT, and p-mTOR/mTOR. (**F**) Quantitative analysis of LC3II/β-actin by densitometry. The data represent the mean ± SD of three independent experiments. Two-way ANOVA; * *p* < 0.05; ** *p* < 0.01; *** *p* < 0.001; **** *p* < 0.0001.

PI3K acts as an initiator of the PI3K-AKT signaling pathway. We used the PI3K agonist 740 Y-P and inhibitor LY294002 to further confirm the role of the PI3K/AKT pathway in V protein-mediated autophagy. The V protein caused LC3-II upregulation after transfection of the Vero cells. After the addition of PI3K activator, the phosphorylation levels of PI3K, AKT, and mTOR were all significantly increased, and the expression level of LC3-II was down-regulated, which inhibited autophagy (Figure 3A,C,D); whereas, after the addition of the inhibitor LY294002, the phosphorylation levels of PI3K, AKT, and mTOR were all significantly decreased, and LC3-II was up-regulated, which enhanced autophagy (Figure 3B,E,F), indicating that CDV-V induced autophagy by inhibiting the PI3K-AKT-mTOR pathway.

### 2.3. Inhibition of PI3K Phosphorylation Facilitates CDV Replication

**Figure 4:**
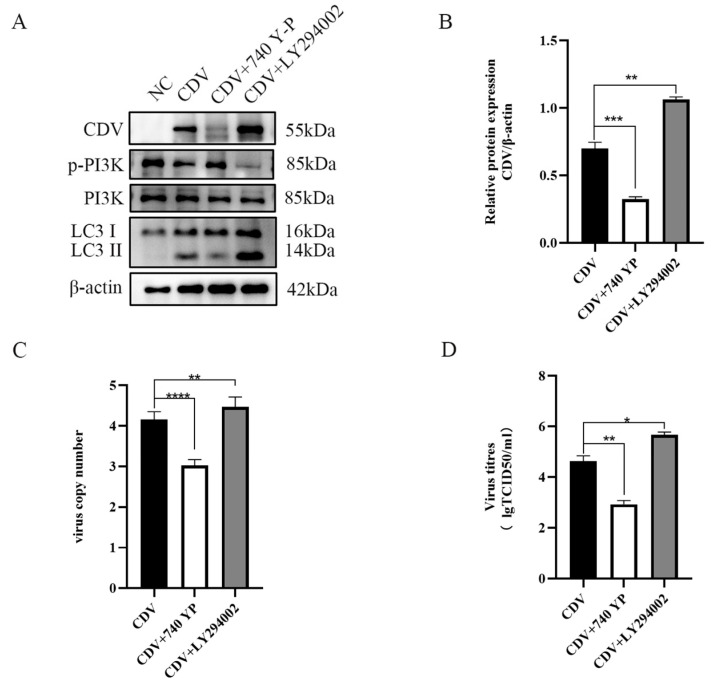
Activation and inhibition of PI3K phosphorylation affects CDV replication. (**A**) Vero cells were pretreated with PI3K agonist 740 Y-P or PI3K inhibitor LY294002 and then infected with CDV; p-PI3K, LC3B, and CDV protein levels were detected by Western blot at 36 h after infection. (**B**) CDV proteins, (**C**) Viral RNA level, and (**D**) virus titer were detected by Western blot, RT-qCPR, and TCID50 36 h after infection, respectively. The data represent the mean ± SD of three independent experiments. Two-way ANOVA; * *p* < 0.05; ** *p* < 0.01; *** *p* < 0.001; **** *p* < 0.0001.

To test the effect phosphorylation of PI3K on viral replication, the PI3K agonist 740 Y-P and inhibitor LY294002 were used to treat the Vero cells and then detect p-PI3K, LC3B, and CDV replication levels at 36 hpi. The results show that compared with the control group, activation of PI3K phosphorylation with 740 Y-P inhibits LC3 II expression and subsequently reduces the CDV protein level (Figure 4A,B), RNA level (Figure 4C), and viral titer (Figure 4D). On the contrary, inhibition of PI3K phosphorylation with LY294002 activates autophagy and promotes viral replication (Figure 4A–D).

**Figure 5:**
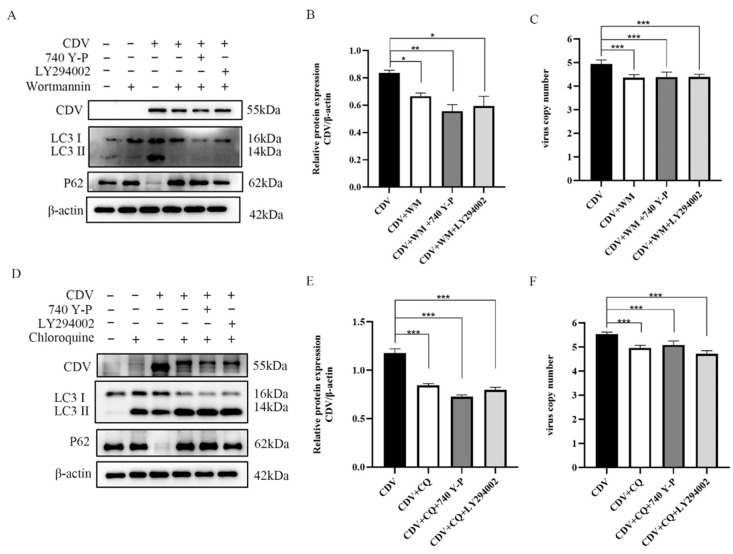
Inhibition of autophagy reduces viral replication. (**A**,**D**) Vero cells were pre-treated with 200 nm Wortmannin and 5 μM Chloroquine, respectively, for 4 h and PI3K activator and inhibitor, then infected with CDV (MOI = 1) for 36 h. The cell samples were then analyzed by Western blot with anti-CDV, anti-LC3B, anti-p62, and anti-β-actin antibodies. (**B**,**E**) CDV protein levels relative to β-actin levels in Wortmannin- and Chloroquine-pre-treated cells were determined by densitometry at 36 hpi. (**C**,**F**) Vero cells were pre-treated and infected as described in (**A**,**B**). At 36 hpi, copy numbers of CDV were measured by qRT-PCR. The data represent the mean ± SD of three independent experiments. Two-way ANOVA; * *p* < 0.05; ** *p* < 0.01; *** *p* < 0.001.

As an important intracellular signaling molecule, PI3K is involved in the regulation of several cellular life processes, such as autophagy, inflammatory response, cell proliferation, and apoptosis [24,25]. In order to determine whether PI3K exerts its effects through autophagy, we treated the cells with Wortmannin (WM) (an early-stage inhibitor of autophagy) and Chloroquine (CQ) (a late-stage inhibitor of autophagy) for 4 h. After this treatment, the cells were treated with PI3K activator or inhibitor, then infected with CDV (MOI = 1) for 36 h. The results showed that the autophagy inhibitor significantly inhibited viral replication, and it could antagonize viral replication triggered by the inhibition of PI3K phosphorylation (Figure 5).

### 2.4. CDV-V Interacts with PI3K

**Figure 6:**
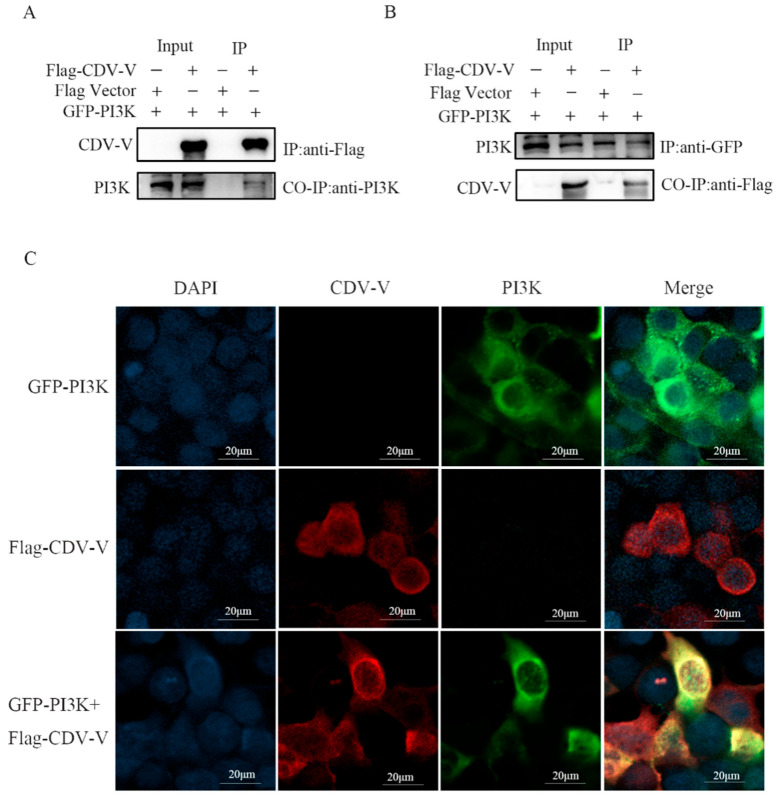
CDV-V protein interacts with PI3K. (**A**,**B**) Flag-tagged CDV-V and GFP-tagged PI3K plasmids were individually transfected or co-transfected into 293T cells. Cell lysate was immunoprecipitated (IP) using mouse anti-Flag or mouse anti-GFP, followed by Western blotting with rabbit anti-Flag and rabbit anti-PI3K. (**C**) 293T cells were transfected individually or in combination with plasmids expressing Flag-tagged CDV-V and GFP-tagged PI3K for 24 h. Cells were fixed and subjected to indirect immunofluorescence analysis using antibodies against Flag-tag (red). The nuclei of the cells were counterstained with DAPI.

To confirm whether the activation of PI3K/AKT is due to the regulation of cellular signaling mechanisms or due to protein–protein interactions between CDV-V and PI3K, Flag-tagged CDV-V protein and GFP-tagged PI3K protein recombinant plasmids were individually transfected or co-transfected into 293T cells for the Co-IP assay. The results of Co-IP showed that CDV-V and PI3K reciprocal specific co-immunoprecipitation was observed with anti-Flag antibody in 293T cells transfected with Flag-CDV-V and GFP-PI3K recombinant plasmids (Figure 6A). Similarly, the presence of CDV-V interactions with PI3K was demonstrated with anti-GFP antibody (Figure 6B).

Further, in order to detect whether CDV-V and PI3K exist co-locally in the cells, GFP-tagged PI3K and Flag-tagged CDV-V plasmid were co-transfected in 293T cells, the cells were incubated with immunofluorescent antibody, and their co-localization was observed using laser confocal microscopy. The results showed that there was co-localization of PI3K and CDV-V in the cells (Figure 6C).

## 3. Discussion

In the replication process of CDV in the cells, the first viral proteins to be produced are the non-structural proteins V and C, which are derived from the P gene through RNA editing [3]. As a crucial protein in the processes of viral evasion of host immunity and viral replication, the paramyxovirus V protein has an additional mechanism for innate immune inhibition beyond its well-known V protein functions, such as MDA5, RIG-I, and STAT antagonism [26,27,28]. Paramyxoviruses could manipulate other immune responses, including apoptosis, stress granules (SGs), and autophagy to promote viral replication. The Mumps virus (MuV) V and Paramyxovirus simian virus 5 (PIV5) V proteins could inhibit caspase 9 and caspase 12, respectively [29,30]. The Newcastle disease virus (NDV) V protein interacts with and downregulates thioredoxin-like protein 1 (TXNL1) or CacyBP/SIP, inhibiting apoptosis and inducing viral replication [31,32]. The V protein of the Measles virus (MeV) could inhibits p53 family member p73 [33]. The human parainfluenza virus type 2 (HPIV2) V protein was found to modulate iron homeostasis and prevent apoptotic cell death, leading to efficient viral growth [34]. The P protein of HPIV3 and the MeV C protein could inhibit SGs [35,36].

Autophagy plays an important role in antiviral and immunity. It can specifically sense microorganisms, such as viruses, that invade cells and promotes the degradation of viruses by autophagic lysosomes. Moreover, autophagy promotes antigen processing and activates the body’s immune response [37,38]. However, an growing number of studies have revealed that many viruses have evolved multiple strategies to hijack and disrupt host autophagy to promote their own replication [39]. Pest des petits ruminants virus (PPRV) can prevent apoptosis through cellular autophagy, thereby enhancing viral replication and maturation in host cells. The PPRV nucleoprotein N and non-structural protein C play an important role in this process by inducing autophagy proteins Agt7 and Beclin-1 to inhibit caspase-dependent apoptosis [40]. MeV induces SQSTM1/p62-mediated mitochondrial autophagy, leading to a reduction in MAVS, which attenuates RLR-mediated activation of the interferon signaling pathway and suppresses the innate immune response [41]. NDV infection-induced autophagy facilitates its replication in cells and tissues [42], and the effect of autophagy on NDV replication is not limited to directly promoting viral replication; it is also associated with a shift in metabolic mechanisms in favor of the virus [43]. The P protein of HPIV3 induces incomplete autophagy by blocking autophagosome–lysosome fusion, resulting in increased virus production [44]. In our previous study, it was demonstrated that CDV can induce autophagy and utilize the autophagy mechanism to promote viral replication. However, the exact molecular mechanism of autophagy induction remains unknown.

When autophagy is formed, the autophagy protein LC3 undergoes a transition from the cytoplasmic form (LC3-I) to the autophagosome membrane-bound form (LC3-II) [45]. The autophagy receptor P62 is located on the autophagosome membrane, and when autophagy is activated, it interacts with LC3 to enter the autophagosome for degradation by lysosomal enzymes [46]. The CDV-V protein is a very important protein in the process of viral evasion of host immunity, as well as viral replication. In order to further investigate the mechanisms of CDV interference with host immunity and the pathway of CDV-induced autophagy, we transfected Vero cells with the recombinant plasmid of the V protein, and the results were the same as those of CDV-infected Vero cells. CDV-V also promotes the up-regulation of LC3, as well as the down-regulation of P62 and induces autophagy.

The PI3K/AKT/mTOR signaling pathway regulates a broad range of cellular processes, including survival, proliferation, growth, and metabolism, and it is also a key pathway in the regulation of autophagy [23]. However, it is still unclear whether CDV induces autophagy through this pathway. In our study, it was found that the CDV-V protein could inhibit the phosphorylated expression levels of PI3K, AKT, and mTOR. To further identify the effect of PI3K on autophagy, we found that when the phosphorylation level of PI3K was activated, it could inhibit LC3-II, and, conversely, inhibition of its phosphorylation could activate LC3-II to antagonize V protein-induced autophagy. In addition, through immunoprecipitation and cellular co- localization, it was determined that the CDV-V protein interacts with PI3K and thus is connected to the PI3K/AKT/mTOR signaling pathway. The exact amino acid sites at which CDV-V interacts with PI3K require further exploration in future studies.

Several members of the *Paramyxoviridae* family, including PPRV [40], MV [41], and NDV [42], can promote replication through autophagy mechanisms. However, the exact molecular mechanism of autophagy induction remains not fully understood. Our study showed that activation of PI3K inhibited the expression of autophagy (Figure 4) and thus inhibited viral replication at the level of viral protein, mRNA, and viral titer, while inhibition of PI3K promoted viral replication compared with the control group. Through further investigation into whether PI3K plays a role in regulating viral replication through autophagy, we found that the ability of PI3K to promote the viral replication was suppressed after the use of the inhibitors of autophagy WM and CQ.

## 4. Materials and Methods

### 4.1. Reagents and Antibodies

The primary antibodies used in this study included antibodies against rabbit LC3B (catalog number 2775), SQSTM1/p62 (catalog number 5114), Phospho-mTOR (Ser2448 and D9C2; catalog number 5536), Phospho-AKT (Ser473 and D9E; catalog number 4060), AKT (catalog number 9272), Phospho-PI3K (Tyr458 and Tyr199; catalog number 4228), and PI3K (19H8; catalog number 4257) were from Cell Signaling Technology, Danvers, MA, USA; β-actin (AF7018, Affinity, San Francisco, CA, USA), Flag (20543-1-AP, Proteintech, Rosemont, IL, USA), mouse monoclonal antibody mTOR (66888-1-lg; Proteintech, Rosemont, IL, USA), CDV-NP (VMRD, Pullman, WA, USA), Flag (F1804, Sigma, Burbank, CA, USA), GFP (66002-1-lg; Proteintech, Rosemont, IL, USA). Secondary antibodies were goat anti-mouse IgG-HRP (31430; Invitrogen, Waltham, MA, USA), goat anti-rabbit IgG-HRP antibody (31460; Invitrogen, Waltham, MA, USA), Alexa Fluor 594 goat anti-rabbit IgG (A11012; Invitrogen, Waltham, MA, USA).

### 4.2. Cell and Virus

African green monkey kidney epithelial cells (Vero) and human embryonic kidney epithelial cells (293T) were preserved in this laboratory. The cells were cultured in Dulbecco’s modified Eagle’s medium (DMEM)(C11995500CP, Gibco, Waltham, MA, USA) supplemented with 10% fetal bovine serum (FBS)(C2510, VivaCell, Shanghai, China), 100 μg/mL penicillin, and 100 μg/mL streptomycin as monolayers in cell culture flasks or dishes at 37 °C under 5% CO_2_.

The CDV3-CL vaccine strain (EU726268) was preserved in our laboratory. The Vero cells were inoculated with CDV and incubated for 2 h. The cells were washed to remove the unbound virus and then cultured in DMEM containing 2% FBS for 72 h. When the cells showed cytopathic changes of about 80%, the cells were frozen and thawed three times, and then the supernatant was taken by centrifugation at 8000 rpm at 4 °C for 10 min. The titer was determined by the Reed–Muench method and expressed as 50% tissue culture infectious dose (TCID_50_).

### 4.3. Plasmids and DNA Transfections

3×Flag-CMV-CDV-V was stored in this laboratory and pcDNA3.1-EGFP-PI3K was purchased from Gene Create (Wuhan, China). For transfection, the Vero cells were cultured in cell culture dishes until 70% fusion. Following the instructions for the Lipo6000™ transfection reagent (C0526; Beyotime, Shanghai, China), 2.5 μg of the 3×Flag-CMV-CDV-V recombinant plasmid and the empty vector 3×Flag-CMV were transfected into the cells. The medium was replaced with fresh medium after 6 h of transfection.

### 4.4. Drug Treatment

The Vero cells were pretreated with or without 10 μM LY294002 (HY-10108; MedChemExpress, Monmouth Junction, NJ, USA) for 1 h; 15 μM of 740 Y-P (HY-P0175; MedChemExpress, Monmouth Junction, NJ, USA) for 3 h; 5 μM Chloroquine (S6999; Selleck, Houston, TX, USA) and 200 nM Wortmannin (S2758; Selleck, Houston, TX, USA) for 4 h, followed by CDV-V recombinant plasmid transfection or CDV infection (MOI = 1). The 740 Y-P is a potent and cell-permeable PI3K activator; it readily binds GST fusion proteins containing both the N- and C- terminal SH2 domains of p85 [47]. LY294002 is a broad-spectrum inhibitor of PI3K class I [48]. Wortmannin is an early-stage inhibitor of autophagy; it inhibits Beclin1/PI3K class III (PtdIns3KC3) autophagy complexes in the upstream of autophagy [49]. Chloroquine is an autophagy inhibitor that can inhibit lysosomal acidification and therefore prevents autophagy by blocking autophagosome fusion and degradation [50].

### 4.5. Western Blotting

The cells were collected and lysed with RIPA lysis buffer (R0020; Solarbio, Beijing, China) supplemented with the protease inhibitor PMSF (P0100; Solarbio, Beijing, China), followed by centrifugation at 16,500× *g* for 10 min at 4 °C to obtain the supernatant. Then, 2 × SDS sample buffer (S3401; Sigma, Burbank, CA, USA) was added to the supernatant and heated at 95 °C for 5–10 min to denature the proteins. Equal amounts of the protein samples were loaded into each well for SDS-PAGE. After the proteins transfer onto the polyvinylidene fluoride (PVDF) membrane, the membrane was blocked at room temperature using 5% non-fat dry milk for 4 h, then incubated overnight at 4 °C in the primary antibody. On the following day, the PVDF membrane was washed three times with TBST for 10 min each. The secondary antibody was incubated at room temperature for 1–2 h, then the membrane was washed three more times with TBST, each for 10 min. Finally, ECL chemiluminescent (KF8001; Affinity, San Francisco, CA, USA) detection solution was used and the results were observed using the BLT GelView6000Plus(Guangzhou Boluteng Biotechnology Company, Guangzhou, China) gel imager.

The working dilution of each antibody used was as follows: in 1000 for the antibodies against p62, PI3K, p-PI3K, p-mTOR, AKT, and p-AKT; in 2000 for the antibodies against LC3B and mTOR; in 5000 for antibodies to CDV-N and β-actin; in 7500 for the antibody to Flag; in 8000 for antibodies to goat anti-mouse IgG-HRP and goat anti-rabbit IgG-HRP.

### 4.6. RT-qPCR

The cells were inoculated in culture dishes, and the viral RNA was extracted using a virus genome DNA/RNA extraction kit (ER201, TransGen Biotech, Beijing, China) according to the manufacturer’s protocol, then reverse-transcribed using a ReverTra Ace qPCR RT Master Mix with gDNA Remover (FSQ-301; Toyobo, Osaka, Japan) into cDNA. Powerup™ SYBR™ Green Master Mix (A25742, Thermo Fisher, Waltham, MA, USA) was used for quantitative real-time PCR. For CDV-specific detection, the CDV-V-F(5′-GCTGACAGTCTCGTGGTA-3′) and CDV-V-R(5′-CAGTTAGATGAAGCATTTCC-3) primer pair was used, and a recombinant plasmid containing the CDV V gene was used to construct a standard curve for calculating the viral RNA copy number in different samples. The specific primers above targeting the conserved region of the CDV V gene were designed using Beacon Designer 8.0 in accordance with MIQE guidelines and were synthesized by Suzhou Hongxun Biotechnology Company Limited (Suzhou, China).

### 4.7. Co-Immunoprecipitation (Co-IP)

293T cells were individually transfected or co-transfected with CDV-V and PI3K expression plasmids for 48 h and Pierce™ Classic Magnetic IP/Co-IP Kit (88804, Thermo Fisher, Waltham, MA, USA) was used for Co-IP. An appropriate amount of cell lysis buffer was lysed at 4 °C for 20 min, then centrifuged at 13,000× *g* for 10 min, and the supernatant was collected. A small portion of the lysate was set aside as a positive control for the input, and the remaining lysate was incubated with 5–10 μg of primary antibody overnight at 4 °C. A total of 25 μL of Pierce Protein A/G magnetic beads was added to the antigen/antibody mixture and incubated at room temperature for 1 h. A magnetic rack was used to collect the beads; they were washed three times and then eluted with elution buffer, and protein interactions were detected using Western blotting.

### 4.8. Immunofluorescence and Confocal Microscopy

The 293T cells were seeded in confocal dishes that had been treated with poly-L-lysine. After the cells were adhered to the confocal dishes, they were individually transfected or co-transfected with the expression plasmids of CDV-V and PI3K incubated for 24 h. The cells were fixed with 4% paraformaldehyde for 20 min, treated with 0.2% Triton X-100 for 15 min, and then blocked with 5% bovine serum albumin for 1 h. The cells were subsequently incubated with the primary antibody overnight at 4 °C, followed by a 1 h incubation in the dark with an appropriate fluorophore-conjugated secondary antibody. Finally, the cells were stained with DAPI (ab104139, Abcam, Cambridge, UK) for 5 min before the samples were imaged using confocal fluorescence microscopy. The 500 dilutions for antibodies against Flag and Alexa Fluor 594 goat anti-rabbit IgG were used.

### 4.9. Statistical Analysis

All the experiments were performed independently three times. Variables were expressed as the mean ± standard deviation (SD) and were analyzed by one-way ANOVA using Graph Pad Prism 8 software. *p* value < 0.05 was considered to be statistically significant (* *p* < 0.05, ** *p* < 0.01, *** *p* < 0.001 and **** *p* < 0.0001).

## 5. Conclusions

In summary, the V protein induces cellular autophagy by interacting with PI3K to inhibit the PI3K/AKT/mTOR signaling pathway, thus inducing autophagy to enhance viral replication. These results improve the understanding of mechanisms of CDV infection and CDV–host cell interactions and provide new insights into the development of effective therapeutic strategies.

## Data Availability

The data that support the findings of this study are available from the corresponding author upon reasonable request.
